# Editorial: Bioaccessibility and bioavailability studies and their importance in the evaluation of health-promoting properties of bioactive compounds

**DOI:** 10.3389/fnut.2023.1157853

**Published:** 2023-02-28

**Authors:** Erick P. Gutiérrez-Grijalva, Marilena Antunes-Ricardo, J. Basilio Heredia

**Affiliations:** ^1^Cátedras CONACYT-Centro de Investigación en Alimentación y Desarrollo (CIAD) A.C., Culiacán, Sinaloa, Mexico; ^2^Tecnologico de Monterrey, Centro de Biotecnología-FEMSA, Monterrey, Nuevo León, Mexico; ^3^Tecnologico de Monterrey, The Institute for Obesity Research, Monterrey, Nuevo León, Mexico; ^4^Laboratory of Functional Foods and Nutraceuticals, Centro de Investigación en Alimentación y Desarrollo (CIAD) A.C., Culiacán, Sinaloa, Mexico

**Keywords:** bioaccessibility, bioavailability, polyphenols, bioactive peptides, terpenoids

In the last decade, it has been observed an increasing demand on the part of consumers to have healthier foods that, in addition to providing nutritional benefits and pleasant sensorial characteristics, can also improve their state of health or prevent the appearance of some diseases. Additionally, there is a strong requirement for high-protein products on the market that can be economical and sustainably produced. This situation has represented important challenges for the food industry, not only for the design and development of new products that contain one or more ingredients considered bioactive compounds but also in the search for new protein sources, the development of new environmentally friendly processes in agreement with the circular economy concept, among many others challenges. This panorama has prompted many researchers to focus their efforts on the search for solutions by studying new novel functional ingredients, and how to incorporate them in the development of new foods.

The study of new potential ingredients based on bioactive compounds must consider very relevant aspects such as their physical and chemical characteristics, as well as their bioaccessibility after digestion, which will undoubtedly determine the most suitable delivery system to ensure their stability, thus guaranteeing greater bioavailability. of the active compounds and their corresponding biological effects.

The study of new potential ingredients based on bioactive compounds must consider relevant aspects of these compounds such as their physical and chemical characteristics, as well as their bioaccessibility after digestion, which will undoubtedly determine the most suitable delivery system to ensure their stability improving their bioavailability of the active compounds and in consequence enhancing their biological effects. As we can understand the different pathways that follow active molecules during their passing through the organism, it will be possible to develop novel products that meet the demands of the market.

This Research Topic is aimed to collect research work related to systemic studies of bioaccessibility, bioavailability, and the bioactive properties of bioactive molecules, as well as the strategies to enhance them in favor of human health. In this Research Topic there are four papers covering the different aforementioned aspects. Ajanaku et al. comprehensively reviews the bioactive and phytochemical constituents in ginger, turmeric, and garlic with an association with their potential pharmaceutical properties against cancer and cardiovascular diseases. Zingiberene and zingerone two of the most studied molecules in ginger have been associated with strong anti-inflammatory and antioxidant properties. On the other hand, curcumin is the main phytochemical found in turmeric, and it's potent anti-inflammatory effect is being studied against inflammatory diseases like arthritis. Lastly, garlic is rich in organosulfur compounds like allicin, allin, and ajoene. Marín-Morales et al. showed that the edible grasshoppers *Sphenarium purpurascens* are a rich source of protein and bioactive peptides with antioxidant properties. Moreover, the authors hypothesize that due to their plant-based feed, grasshopers also contain phenolic acids like protocatechuic acid, hydroxybenzoic acid and flavonoids like luteolin and apigenin. Furthermore, Gong et al. showed the anti-fibrotic effect of extracellular vesicles from tea leaves to liver fibrosis by inhibiting collagen deposition, reducing lipids in the liver, and reduce alanine aminotransferase and aspartate aminotransferase levels. Also, Sharma et al. showed that *Glycyrrhiza glabra* extracts enhance the permeation ov vitamin B12 up to five times *in vitro* and *ex vivo*. Their pharmacokinetic evaluations on Swiss albino mice showed that Cmax, AUC, and Tmax values of B12 were enhanced with the extracts.

## Author contributions

EG-G, MA-R, and JH were responsible for the management of the whole issue. All authors contributed to the article and approved the submitted version.

